# Supplementary material for: Novel Driver Strength Index highlights important cancer genes in TCGA PanCanAtlas patients
Source: PeerJ. 2022 Aug 11;10:e13860. doi: 10.7717/peerj.13860 (PMC9375969; doi:10.7717/peerj.13860)
Supplement: Supplemental Information 2 [file peerj-10-13860-s002.zip › COHORTS/genes plots/detailed/2021_8_16_14_9_distribution_events_detailed_females_11.pdf]

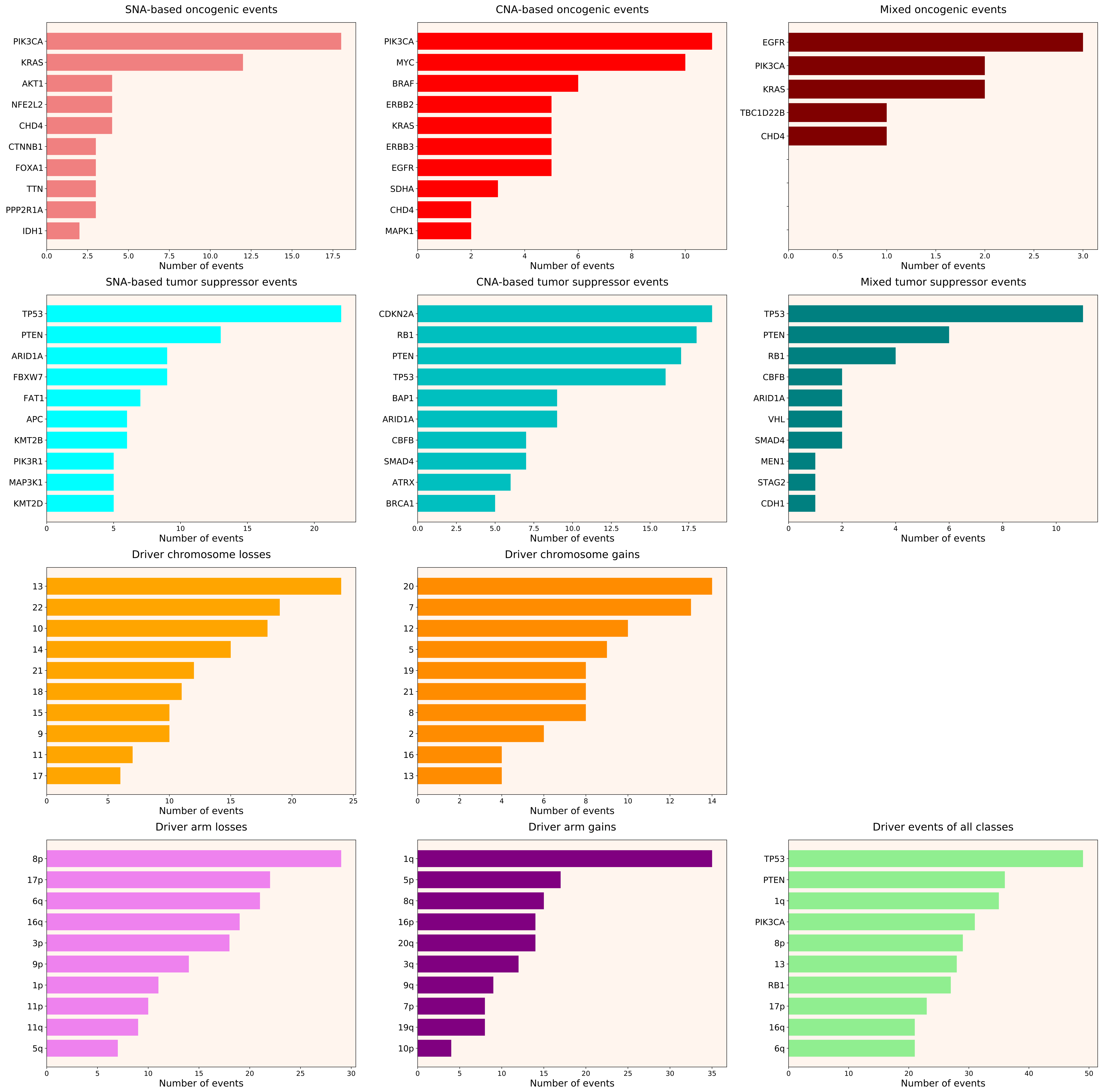

SNA-based oncogenic events
PIK3CA
KRAS
AKT1
NFE2L2
CHD4
CTNNB1
FOXA1
TTN
PPP2R1A
IDH1
0.0 2.5 5.0 7.5 10.0 12.5 15.0 17.5
Number of events
CNA-based oncogenic events
PIK3CA
MYC
BRAF
ERBB2
KRAS
ERBB3
EGFR
SDHA
CHD4
MAPK1
0 2 4 6 8 10
Number of events
Mixed oncogenic events
EGFR
PIK3CA
KRAS
TBC1D22B
CHD4
0.0 0.5 1.0 1.5 2.0 2.5 3.0
Number of events
SNA-based tumor suppressor events
TP53
PTEN
ARID1A
FBXW7
FAT1
APC
KMT2B
PIK3R1
MAP3K1
KMT2D
0 5 10 15 20
Number of events
CNA-based tumor suppressor events
CDKN2A
RB1
PTEN
TP53
BAP1
ARID1A
CBFB
SMAD4
ATRX
BRCA1
0.0 2.5 5.0 7.5 10.0 12.5 15.0 17.5
Number of events
Mixed tumor suppressor events
TP53
PTEN
RB1
CBFB
ARID1A
VHL
SMAD4
MEN1
STAG2
CDH1
0 2 4 6 8 10
Number of events
Driver chromosome losses
13
22
10
14
21
18
15
9
11
17
0 5 10 15 20 25
Number of events
Driver chromosome gains
20
7
12
5
19
21
8
2
16
13
0 2 4 6 8 10 12 14
Number of events
Driver arm losses
8p
17p
6q
16q
3p
9p
1p
11p
11q
5q
0 5 10 15 20 25 30
Number of events
Driver arm gains
1q
5p
8q
16p
20q
3q
9q
7p
19q
10p
0 5 10 15 20 25 30 35
Number of events
Driver events of all classes
TP53
PTEN
1q
PIK3CA
8p
13
RB1
17p
16q
6q
0 10 20 30 40 50
Number of events
